# An overview of RecQ helicases and related diseases

**DOI:** 10.18632/aging.206291

**Published:** 2025-07-25

**Authors:** Tsz-Ching Yiu, Jiajie Tu, Hoi-Hung Cheung

**Affiliations:** 1 School of Biomedical Sciences, Faculty of Medicine, The Chinese University of Hong Kong, Hong Kong S.A.R., China; 2 Centre for Regenerative Medicine and Health, Hong Kong Institute of Science and Innovation, Chinese Academy of Sciences, Hong Kong, China; 3 Institute of Clinical Pharmacology, Anhui Medical University, Key Laboratory of Anti-inflammatory and Immune Medicine, Ministry of Education, Anhui Collaborative Innovation Center of Anti-inflammatory and Immune Medicine, Hefei, China; 4 Key Laboratory for Regenerative Medicine, Ministry of Education, School of Biomedical Sciences, Faculty of Medicine, The Chinese University of Hong Kong, Hong Kong S.A.R., China

**Keywords:** RecQ helicase, DNA repair, maintenance of genome stability, accelerated aging, cellular senescence

## Abstract

RecQ helicases are known as “caretakers” of the genome for their conserved helicase activities to resolve different complex DNA structures. Aberrant accumulation of unsolved DNA structures could lead to defects in DNA replication, gene transcription, and unrepaired DNA lesions. Pathogenic mutations on *BLM*, *WRN*, and *RECQL4* are associated with several pathological conditions, namely Bloom syndrome (BS), Werner syndrome (WS), and Rothmund-Thomson syndrome (RTS). These syndromes are characterized by genomic instability and cancer predisposition. Additionally, some RecQ helicase diseases are linked to developmental defects and premature aging. In this review, we provide an overview of the RecQ helicases, focusing on the molecular functions and mechanisms, as well as the consequences of their dysfunction in cellular processes. We also discuss the significance of RecQ helicases in preventing various genetic disorders (BS, WS, RTS) and the insights obtained from the different animal models developed for studying the pathophysiology of RecQ helicase deficiencies.

## INTRODUCTION

The primary function of DNA helicases is to separate annealed strands in double-stranded nucleic acids and unpack genetic materials within an organism. Coupled with ATP hydrolysis, helicases acquire the free energy needed for nucleic acid separation. By cooperating with other DNA enzymes such as topoisomerases, nucleases, and polymerases, DNA helicases play crucial roles in facilitating DNA repair, replication, transcription, and recombination, thereby ensuring genome integrity and stability.

The RecQ helicase family is conserved across a wide range of organisms, from bacteria and plants to animal kingdoms, indicating its fundamental role in nucleic acid metabolism [1, 2]. In this review, we will focus our discussions on the molecular functions of the three major RecQ helicases, BLM, WRN, and RecQL4, and the related diseases due to their pathogenic mutations.

## RecQ helicase family and its basic function

RecQ helicase was first discovered in an *Escherichia coli* mutant that showed thymineless death (TLD) resistance [3]. This phenotype was caused by the mutation on the r*ecQ* gene, resulting in high sensitivity to UV-induced DNA damage. Further research on bacteria has revealed that this helicase family aids in DNA replication and recovery following DNA damage [4–7]. Subsequently, homologs of the helicase are characterized as “caretakers of the genome” in different species, including *sgs1* in *Saccharomyces cerevisiae*, *wrn-1* in *Caenorhabditis elegans*, and *ffa1* in *Xenopus laevis* [8–11]. As the RecQ family is highly conserved, it suggests a fundamental function of the helicases in DNA metabolism. Further investigations have revealed additional roles of the RecQ helicases in the maintenance of telomere integrity [12]. After the first draft of the human genome was released, five members of the RecQ helicase family were identified and named as *RECQL*, *BLM*, *WRN*, *RECQL4*, and *RECQL5* [13]. Besides their direct role in DNA repair and maintenance of genome stability, RecQ helicase members are also found to regulate aging or aging-associated diseases, which may be an indirect outcome of DNA repair defects [14, 15].

**Figure 1:**
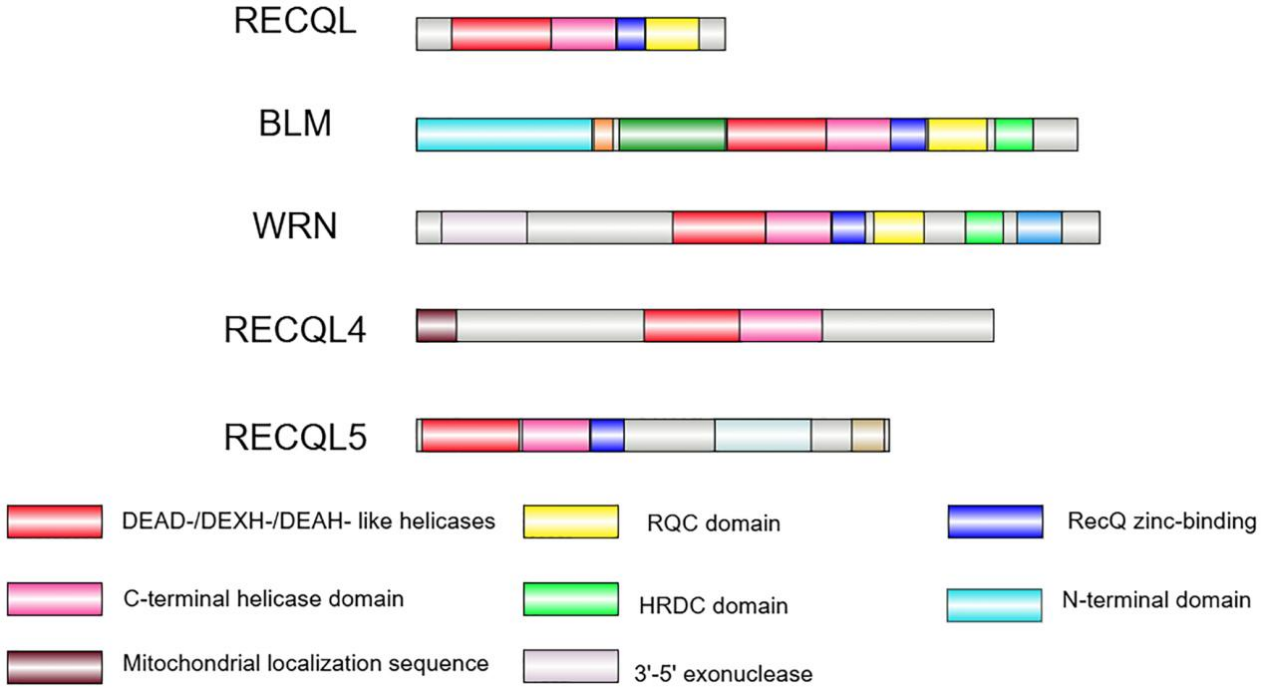
**The RecQ helicase family in human.** The RecQ helicase family contains five members in humans. The helicase core containing a DEAD-/DEXH-/DEAH-like helicase (red box) and a C-terminal helicase domain (pink box) is conserved in all of the family members. The RQC domain (yellow box) is present in RECQL, BLM and WRN helicases. The HRDC domain (green box) is found in BLM and WRN only. The zinc-binding domain (blue box) is conserved in RECQL, BLM, WRN, and RECQL5. The exonuclease domain (purple grey box) is unique to WRN, whereas the N-terminal domain (light blue) is unique to BLM, and the mitochondrial localization sequence (brown box) is unique to RECQL4. Image is created by Illustrator for Biological Sequences (IBS) 2.0.

A notable feature of this family is the presence of a specific RecQ core helicase domain (Figure 1), which contains seven conserved motifs (I, Ia, II, III, IV, V, VI), with an approximate size ranging from 300 to 450 amino acids for each of their helicase domains. The motifs are conserved in the SF1 and SF2 helicase super families, and their common function is to unwind nucleic acids. The core helicase domain normally binds nucleoside triphosphates (NTP) and acquires free energy through NTP hydrolysis, an exergonic process coupled with the helicase activity [16–19]. Some distinct domains, however, are present in specific members only, indicating the unique functions of each RecQ helicase in various molecular interactions. One such unique structure is the RecQ C-terminal (RQC) domain, typically located following the zinc-binding subdomain. The RQC domain is present in RECQL, BLM, and WRN helicases but absent in RECQL4 and RECQL5. Due to its specific folding and sequence, the RQC domain can recognize and bind to DNA duplexes, facilitating the unwinding of double-helical structures [20, 21]. Moreover, the RQC domains in WRN and BLM helicases can recognize and resolve specific DNA secondary structures, such as guanine quadruplex (G4) [22, 23]. Also specific to BLM and WRN helicases, the HRDC (helicase and RNaseD C-terminal) domain is required for interacting with DNA, including single-stranded DNA (ssDNA) and specific double-stranded DNA (dsDNA). It also facilitates protein-protein interactions with different affinities [24–26]. In response to physiological conditions, the HRDC domain increases the helicase activity upon binding to various substrates (e.g., structured nucleic acids) [24, 27]. Because *BLM*, *WRN*, and *RECQL4* have been proven to be associated with well-characterized human genetic disorders that highlight their clinical and biological significance, the following content focuses on these three helicases.

## Molecular function of BLM

BLM helicase (also known as RECQL3) is crucial in maintaining genome stability. BLM has a unique N-terminal domain (NTD) structure that enables it to undergo oligomerization and interact with other proteins. The NTD permits the formation of a large ssDNA loop when resolving duplex DNA. As a result, cells lacking the BLM NTD domain are more sensitive to DNA damage and defective in DNA repair process [58]. This phenomenon could be accounted by the ability of the NTD domain to interact with multiple DNA repair proteins, including RAD51, RAD54, RPA (replication protein A), TP53 (tumor protein p53), RMI1 (recQ-mediated genome instability protein 1), and TOPIIIα (DNA topoisomerase III alpha) [45, 59]. RAD51, RAD54, RPA, RMI1, and TOPIIIα mainly operate within homologous recombination, while RPA and TP53 also participate more broadly in DNA metabolism and damage responses [60–62]. A second structural feature is the HRDC domain near the C-terminus. HRDC is found exclusively in BLM and WRN helicases, although with varied affinities and distinct functions. Interestingly, the HRDC domain not only interacts with proteins but also exhibits a binding preference for ssDNA, suggesting the role of BLM in connecting DNA with other related proteins in the pathway [25].

As a critical component of the DNA repair machinery, BLM helicase plays an important role in choosing homologous recombination (HR) as the preferred repair pathway over non-homologous end joining (NHEJ) and single-strand annealing (SSA) pathways. The HR repair mechanism begins with DNA end resection and homologous strand invasion to extend the invading strand using the homolog as a template [63]. This structure of the dsDNA separated by the invading strand of DNA is named the D-loop. To promote DNA end resection, BLM interacts with DNA2 (DNA replication helicase 2) or EXO1 (exonuclease 1) to generate a 3’ ssDNA tail. The ssDNA tail then recruits RAD51 and forms a presynaptic filament to resolve the D-loop during HR [38, 64, 65]. The physical binding of RPA activates and enhances the unwinding activities of BLM in both nicked and intact ssDNA [66–68]. After the first strand of DNA resynthesis, it can proceed to either the synthesis-dependent strand annealing pathway by dissociating the newly synthesized strand, or to the dsDNA break (DSB) repair pathway by capturing the second strand of DSBs for another resynthesis [69].

To ensure proper maintenance of the genome, the selection of the DNA repair pathway (NHEJ vs. HR) depends on the cell cycle stage [70, 71]. It is demonstrated that BLM is essential for recruiting classic NHEJ factors during the G1 phase and HR factors during the S phase. BLM can also act as a negative regulator that inhibits HR in the S phase and classic NHEJ in the G1 phase. The inhibition of BLM breaks the balance between repair pathways [72]. In addition to the classical NHEJ discussed above, BLM is also capable of inhibiting the alternative NHEJ pathway, which is a primary cause of highly error-prone chromosomal translocations [73]. BLM does so by protecting DNA from the attachment of CtIP/Mre11 and promoting the aggregation of 53BP1 (p53-binding protein 1) at DSBs [74].

During the DSB repair pathway, a complex DNA structure known as the double Holliday junction (dHJ) is formed to prevent crossing-over, thereby avoiding genomic instability and chromosomal rearrangements. By interacting with TOP3a and RMI1/RMI2, BLM can form a BLM dissolvasome (also known as the BTR complex) to resolve the dHJ [75]. Notably, in patients with “Bloom-like syndrome”, genetic mutations of RMI or TopIIIα were found, suggesting the phenotypes of BS may result from the dysfunction of the BTR complex [76, 77].

In addition to DNA repair, BLM also participates in DNA replication. A replication fork is a three-way junction between replicated and unreplicated portions of a DNA molecule. The progression of replication forks can be stopped by DNA damage, secondary nucleic acid structure, and protein-DNA complex [78]. The helicase domain in BLM specifically recognizes and resolves the DNA structures in a wide range of DNA substrates, including G4, D-loop, dHJ, and forked duplexes (Table 1) [38, 44, 79, 80]. Along with its role in the HR pathway and physical interactions with RPA, BLM can stabilize replication and remodel it to restart the replication process [77, 81–83]. Concomitantly, sumoylation, the post-translational modification that covalently attaches small ubiquitin-like modifier (SUMO) to target proteins, serves as another important mechanism through which BLM influences DNA replication. BLM sumoylation is essential for the stability and restart of replication forks, particularly under replication stress, as its absence leads to reduced fork velocity, increased fork collapse—a process where the replication fork structure disintegrates—and impaired recruitment of RAD51 to stalled forks [84]. Through sumoylation regulation, the function of BLM is switched between pro- and anti-roles in HR by regulating the localization of RAD51 at damaged replication forks, thereby determining whether the stalled replication forks are restarted [85]. Telomeric replication is a subtype of replication that poses a challenge for the replication machinery due to the G-rich tandem repeats. The maintenance of the telomere length relies on telomerase and alternative lengthening of telomeres (ALT) [86]. BLM helicase plays a significant role in telomeric replication by resolving replication fork-blocking G4 structures and correctly dissociating telomeric regions from the BTR complex in the ALT pathway [87]. Notably, the RQC domain is responsible for resolving some repetitive structures like telomeric G4. The G4 resolving activity is an important function of BLM and WRN within the RecQ helicase family. In general, BLM plays a versatile role in DNA repair that goes beyond simply binding to DNA. By concluding the function and selection of BTR complex reviewed by Manthei and Keck [88], and the summary of BLM’s role in the initiation and maintenance of ALT presented by Chang and their team [89], the complexity of how BLM maintains genome stability is well illustrated.

**Table 1:** The ability of the RecQ helicases to unwind DNA/RNA structures.

	**Cruciform DNA** 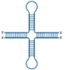	**Double strand helix** 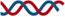	**D-Loop** 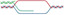	**G-Quadruplex (G4)** 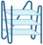	**Holliday junction** 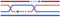	**R-Loop** 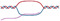
RECQL1		✓ [28, 29]		✓ [30–32]	✓ [28, 33]	
BLM	✓ [34]	✓ [35–37]	✓ [38–40]	✓ [20, 41–44]	✓ [45–47]	✓ [48, 49]
WRN	✓ [34]	✓ [37, 50]	✓ [23, 44, 51]	✓ [23, 41]	✓ [23, 52]	✓ [49, 53]
RECQL4		✓ [54, 55]				
RECQL5		✓ [37, 56]			✓ [36, 37, 57]	

One remarkable feature in cells lacking BLM helicase is the loss of heterozygosity and increase in sister chromatid exchange (SCE). SCE is a byproduct of the DNA repair pathway triggered by DSB or a collapsed replication fork formed during HR. Hence, the SCE level serves as an index for assessing chromatin instability and HR deficiency [90–92]. As discussed above, BLM’s ability to dissolve dHJ and regress replication forks directly contributes to maintaining chromosome stability and preventing SCE. Interestingly, sequencing the SCE genomic locations of BLM-deficient cells reveals that SCEs are not randomly distributed across the genome but are enriched explicitly in coding regions, particularly at locations with G4 motifs in the transcribed genes [93]. Generally, cells with BLM deficiency tend to experience failures in dHJ dissolution, increased recombination in G4, and impaired replication fork management, all of which can lead to SCE.

Besides safeguarding DNA replication, BLM also aids in transcriptional regulation by resolving secondary structures and interacting with transcription factors. Since more than 40% of human gene promoters contain one or more G4 structures, BLM likely plays a crucial role in transcription by modulating the accessibility of these promoters to transcription factors (TFs) [94]. In the differential expression of mRNA between wild-type and BLM-depleted cells, G4 motifs exhibit significant enrichment at transcription start sites (TSSs) and are particularly concentrated within the first intron [95]. This regulation of TSS structure may influence gene expression by altering the chromatin accessibility of transcription factors. Furthermore, BLM can directly interact with c-Jun and RNA polymerase, affecting their binding efficiency to gene promoters, thereby facilitating or inhibiting transcription [96, 97]. Lastly, BLM further regulates transcription by resolving R-loops, which are secondary structures formed by the hybridization of nascent RNA to its complementary DNA template, thereby protecting the actively transcribed sites from DNA damage [48, 98]. Removing R-loops improves transcription by preventing conflicts between transcription and replication.

In conclusion, BLM can play multiple roles in maintaining genome stability by participating in DNA damage repair, replication, and transcription. It alters the dynamics of the DNA repair pathway in each cell cycle phase to ensure low error rates. It restarts stalled replication forks and resolves secondary nucleic acid structures such as G4, D-loop, and R-loop. Furthermore, BLM regulates transcription by modifying the chromatin accessibility and the recruitment of transcription factors. Overall, BLM helicase is a critical safeguard of genome integrity.

## Molecular function of WRN

The *WRN* gene encodes the WRN helicase and is usually mutated in Werner syndrome (WS) [99]. Similar to BLM helicase, WRN helicase contains the core helicase domain in addition to the RQC domain and HRDC domain. Notably, WRN is the only member of the RecQ helicase family that contains an exonuclease domain in addition to the helicase domain, making it a large multifunctional protein [15]. The exonuclease has an activity specific to the 3’-to-5’ direction. The exonuclease domain is located near the N-terminus, allowing it to resolve various DNA structures, including flap-structured dsDNA, bubble structure duplex, and fork-shaped duplex. WRN and BLM can interact and cooperate with each other; and not surprisingly, they share many common binding partners, such as DNA2, EXO1, RAD51 and RPA [100–102]. Critically, the helicase activity of WRN is weak when it is present solely. Under the stimulation by RPA, WRN becomes a powerful helicase that can unwind more than 1 kb of dsDNA unidirectionally [103].

Being an essential member in maintaining genome stability, WRN functions in optimizing the DNA repair pathway by playing various roles in HR, classic NHEJ, alternative NHEJ, and SSA. The absence of WRN was found to result in accumulated DNA damage, a lower proliferation rate, loss of epigenetic marks, and premature senescence [104–107]. Considering that these WRN-deficient cells are also sensitive to DNA damage-inducing agents [108–110], the impairment of DNA repair is one of the critical reasons.

Independent of the recruitment of DNA repair proteins, WRN can be directly recruited to and accumulate at the DSB sites via its HRDC domain, enabling persistent and rapid recruitment to stabilize DSBs [111]. Furthermore, the phosphorylation of WRN, which might be cell-phase dependent, alters its affinity to RPA and results in the selection of the repair pathway. If DSBs are present in the G1 phase, WRN is unphosphorylated on S426 and has a lower affinity toward RPA. In case DSBs are present in the S or G2 phases, S426 of WRN would be phosphorylated by active CDK2, increasing WRN’s affinities for RPA and RAD51 to promote strand invasion and D-loop formation [112]. The interaction between WRN and Ku70/Ku80 complex specifically enhances the exonuclease activity of WRN [113]. The Ku heterodimer is recruited to the DNA ends of DSBs. By interacting with the Ku complex and DNA-PKcs, WRN inhibits the recruitment of the MRN (MRE11/RAD50/NBS1) complex, which is known as a DNA binder, to the DSB ends [114, 115]. Furthermore, it suppresses the recruitment of MRE11 and CtIP, thereby preventing the initiation of alternative NHEJ. The binding of WRN in DSBs promotes classic NHEJ through its helicase and exonuclease activities while inhibiting alternative NHEJ through non-enzymatic functions, thereby increasing the affinity for classic NHEJ [116]. Following WRN’s enzymatic action, the DNA ends are trimmed to overhangs, becoming a form that is suitable for the ligation initiated by the XRCC4-DNA ligase IV complex, and thus promoting classic NHEJ [117]. WRN in this pathway is a regulator to choose between classical and alternative NHEJ depending on the microhomology [116].

Additionally, WRN and BLM are the only helicases in the RecQ helicase family that can resolve G4 structures. As discussed above, G4 structures are typically found in telomeres and promoters. By comparing the transcriptional output between normal and WS fibroblasts, a significant enrichment of the computational G4 motif was found downregulated in WS fibroblasts [118]. Consistently, our lab specifically identified a G4 substrate for WRN in the regulation of short stature through unwinding the *SHOX* (short stature homeobox protein) promoter G4 and modulating its transcription [119]. WRN might also affect the abundance of G4 indirectly by aiding the G4 processing activity of WRNIP1 (Werner helicase interacting protein 1) [120].

In conclusion, WRN is a multifunctional helicase within the RecQ helicase family, distinguished by unique molecular features that differentiate it from BLM, which is functionally and structurally similar to WRN in general. WRN’s specific exonuclease activity enables it to detect and process different DNA structures effectively, playing a crucial role in DNA repair and maintaining genome stability. Through interactions with diverse protein partners, including RPA, Ku70, and WRNIP1, WRN participates in critical DNA repair pathways (such as NHEJ) and plays a decisive role in regulating replication stress responses. The ability of WRN to resolve G4 structures highlights its specialized function, particularly in telomere maintenance and gene regulation.

## Molecular function of RECQL4

*RECQL4* is the gene mutated in Rothmund-Thomson syndrome (RTS). Like BLM and WRN, RECQL4 is also involved in DNA repair, recombination, and replication. It was first found and characterized by Kaito and their team in 1998, together with another RecQ member RECQL5 [121]. Unlike RECQL1, BLM, and WRN, which are nuclear proteins, RECQL5 is found in both the nucleus and cytoplasm, whereas RECQL4 is the only member found in not only the nucleus and cytoplasm but also in the mitochondrion. Contrasting with WRN and BLM, RECQL4 lacks the HRDC and RQC domains, which are thought to be the putative DNA binding domains. Additionally, RECQL4 is shown to have intense DNA annealing activity [122]. As a helicase member, RECQL4 also demonstrates the helicase activity by helicase assays [123]. RECQL4 can promote NHEJ by interacting with Ku70 to repair DSBs. RECQL4 activity is regulated by its phosphorylation and ubiquitination. The phosphorylation enhances the helicase activity and increases HR efficiency to promote cell survival [124]. Through interacting with different partners, REQCL4 shows distinct functions in different molecular processes. For example, it can interact with SLD5 and MCM7 that participate in DNA replication. In other scenarios, RECQL4 interacts with telomere-binding proteins TRF1 (telomeric repeat binding factor 1) and TRF2 (telomeric repeat binding factor 2) to regulate telomere stability [51, 125]. Although RECQL4 is not able to resolve G4 structure, it was shown to play a role in telomere maintenance by associating with shelterin proteins and resolving telomeric D-loop structure by synergetic interaction with WRN [51].

Different from most of the proteins involved in DNA repair pathway, RECQL4 has a unique function in mitochondrial genome maintenance and crosslink repair. The N-terminus of RECQL4 has a mitochondrial localization signal (MLS) domain that enables it to shuttle into the mitochondria. By co-transporting p53, RECQL4 participates in the replication of mitochondrial genome [126]. In cells with RECQL4 deficiency, accumulation of mitochondrial DNA (mtDNA) damage is observed [127]. RECQL4 thus serves as an accessory factor for the initiation and regulation of mtDNA replication, which is crucial for maintaining the mtDNA copy number [128].

At the cellular level, cells deficient in RECQL4 exhibit a higher propensity for chromosome mosaicism, increased apoptosis, and an accumulation of mitotic irregularities. This tendency is primarily attributed to a higher ratio of cells that become trapped during the prophase of mitosis [129–131]. Similar to the effects seen with the depletion of BLM and WRN, cells lacking RECQL4 also exhibit increases in senescence signals and accumulated DNA damage [132]. Research involving RECQL4-deficient cell lines has demonstrated significant alterations in mitochondrial function and dynamics due to the role of the MLS domain. The absence of RECQL4 in these cells results in a marked reduction in mtDNA copy number, an increase in ROS level, and a disruption in mitochondrial DNA repair pathway. Additionally, these cells exhibit a notable decrease in mitochondrial bioenergetic capacity, along with increased fragmentation of mitochondria [127, 128, 133].

To summarize, RECQL4 is a helicase that plays a significant role in DNA repair, recombination, and replication, particularly in mitochondrial function. The MLS domain enables RECQL4 to enter mitochondria and regulate mtDNA replication. Although it is structurally different from BLM and WRN, it also participates in the maintenance of genome stability in NHEJ, HR, and BER (base excision repair) pathways through other interactions and its own helicase activities.

## BLM dysfunction and bloom syndrome

BLM dysfunction is directly linked to an autosomal recessive genetic disorder “Bloom syndrome” (BS). The affected individuals typically present with prenatal and postnatal growth retardation, sun-sensitive skin lesion, poikiloderma, immunodeficiency, increased risk of diabetes, and cancer [134–138]. The growth retardation of BS patient starting prenatally results in the small for gestational age, shorter birth length and lower birth weight (Table 2) [135, 139, 140]. Throughout childhood and into adulthood, individuals affected by this condition remain significantly shorter and lighter than their peers, typically measuring around 15–20% below standard height and weight norms [135]. The cells isolated from BS and the cells with BLM deficiency exhibit increased reactive oxygen species (ROS) and oxidative DNA damage, which may lead to a reduction in the DNA replication rate and impaired cell cycle progression [141–144]. The dysregulation of cellular processes, which negatively affect the proliferation pathway, results in a decreased proliferation rate, leading to a growth delay in BS [141].

**Table 2:** Clinical signs and symptoms for RecQ deficiency diseases [231–234].

	**Werner syndrome**	**Bloom syndrome**	**Rothmund-Thomson type 2**	**Baller-Gerold syndrome**
**Very frequent**	Abnormal hair whorl	Abnormality of the immune system	Erythema	Aplasia/Hypoplasia of the radius
	Abnormal thorax morphology	Adipose tissue loss	Hyperpigmentation of the skin	Aplasia/Hypoplasia of the thumb
	Abnormality of the voice	Decreased circulating antibody level	Hypopigmentation of the skin	Brachycephaly
	Cataract	Growth delay	Poikiloderma	Brachyturricephaly
	Convex nasal ridge	Intrauterine growth retardation		Failure to thrive in infancy
	Hypogonadism	Severe postnatal growth retardation		Frontal bossing
	Lipoatrophy	Small for gestational age		Growth delay
	Osteoporosis			Hand oligodactyly
	Pili torti			Large fontanelles
	Premature graying of hair			Proptosis
	Prematurely aged appearance			Short stature
	Short stature			
	Slender build			
	Sparse scalp hair			
	White forelock			
**Frequent**	Abnormal testis morphology	Abnormal proportion of CD8 T cells	Abnormality of the dentition	Abnormal carpal morphology
	Abnormality of retinal pigmentation	Cafe-au-lait spot	Aplasia/Hypoplasia of the eyebrow	Abnormal metacarpal morphology
	Aplasia/Hypoplasia of the skin	Cutaneous photosensitivity	Dermal atrophy	Anteriorly placed anus
	Aplasia/Hypoplasia of the testes	Decreased circulating IgA level	Facial erythema	Aplasia/Hypoplasia of the patella
	Atherosclerosis	Decreased circulating IgG level	Growth delay	Bowing of the long bones
	Chondrocalcinosis	Decreased circulating total IgM	Multiple skeletal anomalies	High palate
	Congestive heart failure	Decreased head circumference	Nail dysplasia	Intrauterine growth retardation
	Decreased fertility	Decreased proportion of CD4-positive T cells	Short stature	Malabsorption
	Hyperkeratosis	Gastroesophageal reflux	Small for gestational age	Narrow mouth
	Increased bone mineral density	Hypopigmentation of the skin	Sparse hair	Short nose
	Insulin resistance	Insulin resistance	Sparse or absent eyelashes	
	Lack of skin elasticity	Malar flattening		
	Lipodystrophy	Male infertility		
	Myocardial infarction	Micrognathia		
	Narrow face	Narrow face		
	Pulmonary artery stenosis	Neoplasm		
	Rocker bottom foot	Otitis media		
	Skeletal muscle atrophy	Poor appetite		
	Skin ulcer	Premature ovarian insufficiency		
	Small hand	Recurrent infections		
	Subcutaneous calcification	Retrognathia		
	Telangiectasia of the skin	Skin rash		
	Type II diabetes mellitus			
**Occasional**	Abnormality of the cerebral vasculature	Abdominal obesity	Abnormal blistering of the skin	Abnormal cardiac septum morphology
	Acral lentiginous melanoma	Abnormal blistering of the skin	Abnormal trabecular bone morphology	Abnormal localization of kidney
	Breast carcinoma	Abscess	Abnormality of dental enamel	Abnormality of the ureter
	Cutaneous melanoma	Acute lymphoblastic leukemia	Abnormality of immune system physiology	Anal atresia
	Gastrointestinal carcinoma	Acute myeloid leukemia	Abnormality of the radial head	Broad forehead
	Hypertension	Azoospermia	Abnormality of ulnar metaphysis	Cleft palate
	Joint stiffness	Bronchitis	Alopecia totalis	Conductive hearing impairment
	Laryngomalacia	Cheilitis	Anemia	Epicanthus
	Melanoma	Chronic pulmonary obstruction	Aplasia/hypoplasia involving bones of the upper limbs	Hydronephrosis
	Meningioma	Diabetes mellitus	Basal cell carcinoma	Hypertelorism
	Neoplasm	Gastrostomy tube feeding in infancy	Carious teeth	Hypotelorism
	Neoplasm of the lung	Lymphoma	Cleft palate	Lymphoma
	Neoplasm of the oral cavity	Malignant genitourinary tract tumor	Cryptorchidism	Micrognathia
	Neoplasm of the small intestine	Myelodysplasia	Delayed eruption of teeth	Narrow face
	Ovarian neoplasm	Neoplasm of the breast	Delayed skeletal maturation	Narrow nasal bridge
	Renal neoplasm	Neoplasm of the colon	Developmental cataract	Nystagmus
	Sarcoma	Neoplasm of the skin	Diarrhea	Osteosarcoma
	Secondary amenorrhea	Oligozoospermia	Facial edema	Poikiloderma
	Spontaneous abortion	Paronychia	Functional abnormality of the gastrointestinal tract	Prominent nasal bridge
	Squamous cell carcinoma	Patchy alopecia	Genu varum	Scoliosis
	Thyroid carcinoma	Pneumonia	High palate	Urogenital fistula
		Poikiloderma	Joint dislocation	Vesicoureteral reflux
		Recurrent gastroenteritis	Long nose	
		Recurrent herpes	Lymphoma	
		Recurrent tonsillitis	Metaphyseal sclerosis	
		Recurrent urinary tract infections	Metaphyseal striations	
		Respiratory tract infection	Microdontia	
		Rhinitis	Myelodysplasia	
		Severe toxoplasmosis	Nasogastric tube feeding	
		Severe varicella zoster infection	Neoplasm of the skin	
		Sparse eyelashes	Neutropenia	
		Telangiectasia	Osteopenia	
		Uveitis	Osteosarcoma	
			Patellar aplasia	
			Patellar hypoplasia	
			Pathologic fracture	
			Plantar hyperkeratosis	
			Reduced number of teeth	
			Short metacarpal	
			Short phalanx of finger	
			Slender nose	
			Squamous cell carcinoma	
			Synostosis involving bones of the upper limbs	
			Vomiting	

One of the clinical features of BS that has raised the most attention from researchers is immunodeficiency. Schoenaker and their team compared the blood samples of BS patients and found that they have lower serum immunoglobulin levels and reduced T cells, B cells, and NK cells, while having a specifically higher percentage of CD4+ and CD8+ effector memory T cells [145]. The critical role of BLM helicase in the differentiation and functional maintenance of the αβ T-cell lineage helps explain the immunodeficiency observed in BS patients. BLM is essential for not only the early T-cell differentiation but also for the ability of thymocytes to receive the beta selection signal. Consequently, the decreased numbers of T cells and thymocytes in *Blm* conditional knockout mice are a result of impaired proliferation and survival within the immune cell signaling pathways [144].

From the view that BS patients have high tendency to develop various types of cancer, BLM is regarded as a tumor suppressor. The ability of BLM to facilitate the degradation of the oncogene transcription factor c-Jun has been previously demonstrated, supporting the tumor-suppressing role of the BLM helicase [97]. This degradation mechanism alters the function of c-Jun to activate its downstream oncogenes, so it partially explains the increased cancer susceptibility and genomic instability observed in BS patients. Moreover, *Blm* mutation in mice with *Ptch* (patched 1) deficiency (*Ptch1*^+/−^*Blm*^tm3Brd/tm3Brd^) develops tumors more aggressively than *Ptch1*^+/−^*Blm*^+/+^ mice. Chromosome aneuploidy is associated with loss of p53 as a result of genomic instability in these mutant mice [146]. In other studies on human subjects, the heterozygosity of BLM increases the risk and progression of colorectal cancer and breast cancer [147].

While simply defining BLM as a “tumor suppressor” may not truly reflect its actual role in normal and cancer cells. BLM expression is associated with cell proliferation and differentiation. In non-neoplastic cells, BLM is highly expressed in actively proliferative cells such as epithelial cells of the digestive tract and the skin. The high expression of BLM in undifferentiated cells and progenitors, and with the evidence that BLM suppresses the expression of certain differentiation markers, suggest that BLM may promote proliferation and inhibit differentiation [148]. In some cancers, however, BLM is required for their uncontrolled proliferation. The expression of BLM is high in cancers such as lymphoma and carcinomas originated from prostate and epithelium [149, 150]. The function of BLM in tumorigenesis and tumor progression could be explained by the inhibition of apoptosis and promotion of proliferation in prostate cancer but not in normal prostate tissue [151]. Prostate cancer is not the only malignancy associated with increased BLM expression; similar patterns have been observed in patient samples and cell lines from colon and breast cancers [152–154]. Additionally, in prostate cancer patients receiving chemotherapy or immunotherapy, BLM expression levels have been found further increased [155]. As a result, scientists have explored the possibility of targeting BLM to improve survival rate in breast, colon, and prostate cancers. Combining chemotherapy and/or immunotherapy with BLM inhibitors, the improved survival rates observed provide further evidence of BLM’s role in cancer progression [156].

Nuclear cataracts, a subtype of age-related cataracts (ARC), are another clinical feature shown to be associated with the pathological mutation of *BLM* [157]. The relationship between BLM and cataract progression has been identified as being linked to its regulation of the capsular lens cell viability and apoptosis. Notably, knockdown of BLM protein accelerates the progression of ARC, and this effect is further exacerbated by UV stimulation [158]. Additionally, characteristics of photosensitive skin indicate that BS patients’ skin reacts more strongly to UV radiation from sunlight. A deficiency in BLM, a crucial DNA repair helicase, reduces the effectiveness of DNA damage repair caused by UV radiation, providing an indirect explanation for the photosensitive skin [159, 160].

While many early important findings came from *in vitro* studies and cell-based experiments, investigating the function of the *BLM* gene in a complex organism could help elucidate the disease phenotype observed in BS and clarify its paradoxical role in tumor suppression or promotion. Chester and their team generated complete *Blm* knockout mice. They found developmental delay and embryonic lethality before E13.5 [161]. Although *Blm* knockout cells also showed a high frequency of SCE similar to human cells, the embryonic lethality in this strain limited the study of disease pathogenesis. Conversely, Luo’s group successfully created viable *Blm^m3^* knockout mice with premature termination of the protein. In these mice, increased loss of heterozygosity and SCE were observed. Moreover, they also observed cancer predisposition and accelerated methylation aging in multiple tissues [162, 163]. Apart from mammals, several groups also established zebrafish model to study BLM function. Interestingly, the deletion of *blm* in zebrafish resulted in germ cell differentiation problem, in which only male zebrafish could develop following sexual determination. The homozygous mutant fish also showed reduced fertility (and longevity) due to aberrant spermatogenesis with meiotic arrest [164, 165]. These studies recapitulate some of the features in BS patients and suggest a conserved function of the BLM helicase in safeguarding gametogenesis during sexual differentiation. Together with the mouse and human iPSC models [166, 167], these studies suggest a vital role of BLM helicase in normal development and prevention of diseases like cancers.

## WRN dysfunction and Werner syndrome

The WRN helicase is associated with WS, an autosomal recessive disease characterized by premature aging. WS was first described in a family with four siblings, aged between 31 and 40, who exhibited symptoms such as premature graying of hair, scleroderma cataracts, and short stature [168]. The gene associated with WS was later mapped to a member of the RecQ helicase family. Despite its rarity, WS draws the attention of gerontologists because of its phenotypes of accelerated aging in patients. Usually, the first sign of diagnosis is the lack of growth spurt at puberty and short stature. After puberty, patients exhibit early onset of premature aging phenotypes as early as the 20s to 30s, such as baldness, graying of hair, loss of subcutaneous fat, and atrophy of muscle and skin [169]. Other symptoms include foot ulcers, cataracts, atherosclerosis, osteoporosis, type II diabetes, and malignancy, which become more frequent in the middle age of patients (Table 2) [170]. Because of the high incidence of developing myocardial infarction and malignancy, the median age of WS patients is approximately 54, which is generally shorter than normal longevity [171].

**Figure 2:**
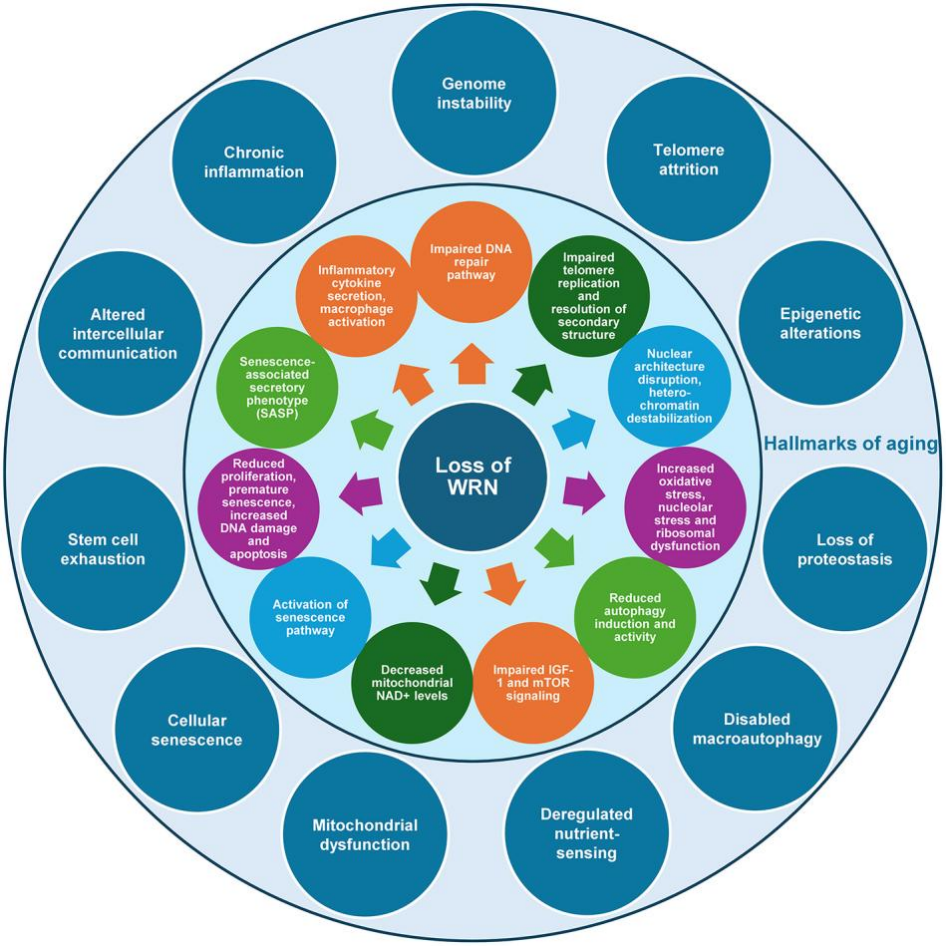
**Conceptual framework illustrating how loss of WRN leads to the hallmarks of aging.** Loss of WRN helicase results in alterations in different cellular and molecular processes (circles in the inner zone). These processes are linked to the known hallmarks of aging (circles in the outer zone) [173]. For instance, impaired DNA repair is associated with genome instability, whereas reduced proliferation and premature senescence are connected to stem cell exhaustion.

An expanding body of research aims to elucidate the relationship between the molecular functions of WRN helicase and the clinical and pathological features of WS (Figure 2). While cataracts are frequently observed in patients with WS, a decrease in the expression of the *WRN* gene due to epigenetic factors has been noted in the anterior lens capsules of ARC patients [172]. Epigenetic alteration is one of the hallmarks of aging [173]. Notably, epigenetic reprogramming by Yamanaka factors is known to reset the epigenetic status in differentiated cells as well as in senescent cells. We and others demonstrated that when WS iPSC was differentiated into mesenchymal stem cells (MSC), premature senescence recurred [174]. A similar phenomenon was observed in the MSCs derived from WRN-deleted embryonic stem cells (ESC) [175, 176]. Epigenetic marks, including DNA methylation, histone modification, chromatin remodeling, and RNA modification, are copied and passed to the daughter cells during replication. However, due to increased ROS production and other random DNA damage, some epigenetic marks are lost during cell proliferation or senescence [177]. Some specific epigenetic modifications are now defined as age-associated markers, such as the age-associated variably methylated positions (aVMPs), and the global loss of H3K9me3 heterochromatin. Consistently, these chronological markers are also altered in WRN-deficient cells, demonstrating the function of WRN in preventing stem cell senescence [176].

In the absence of WRN, heterochromatin is destabilized with the loss of epigenetic modification complexes. The loss of heterochromatin and the scaffold protein HP1α (heterochromatin-binding protein 1α) results in the disorganization of nuclear architecture, genome instability, and cellular senescence. Zhang and their team found a marked decrease in the abundance of H3K9me3 heterochromatin, accompanied by a reduction in heterochromatin architecture at the nuclear periphery in WRN-null cells [176]. The deficiency of WRN results in the instability of the functional complex formed with the histone methyltransferase SUV39H1, HP1α, and nuclear lamina-heterochromatin anchoring protein LAP2β, consequently leading to impaired establishment and maintenance of H3K9me3-enriched heterochromatin domains [176]. A substantial body of evidence from both experimental models and human studies demonstrates that a decrease in H3K9me3 is a hallmark of cellular and organismal aging, with losses observed not only in WRN-deficient cells but also in physiologically aged tissues, progeroid syndromes, and across various somatic cell types [178–181]. Sidorova and their group further prove this mechanism of WRN-dependent heterochromatin maintenance by identifying the association of WRN with histone deacetylase HDAC2 and HP1α as a complex, interacting with lamin B1 (LMNB1) and the lamin B receptor (LBR). This model elucidates the loss of peripheral heterochromatin observed in WRN deficiency cells, coinciding with the general epigenetic hallmark of aging [182].

Tsuge and their team have categorized the molecular phenotypes of WS into several types of dysfunction: transcriptional dysregulation, repair dysfunction, chromosomal instability, stem cell senescence, mesenchymal cell senescence, endothelial cell senescence, and telomere dysfunction [183]. These markers have made WS patient cells a valuable model for studying cellular senescence and accelerated aging in gerontological research. Classically, studies on cellular aging involve comparing gene expression changes between aged and young fibroblasts, and these transcriptional changes are highly recapitulated in WS cells [184]. The aging-related symptoms seen in various organs and tissues, such as the skin, cornea, and bone, suggest that the WRN helicase plays a specific role in preventing senescence in particular cell types. The signature symptoms of WS include short stature, osteoporosis, skin atrophy, loss of fat tissue, and muscle wasting. These phenotypical analyses indicate that the tissues affected in WS patients primarily originate from mesenchymal lineages. It also suggests that loss of WRN helicase may specifically affect certain cell types and tissues. Reduced proliferation, premature senescence, and increased DNA damage and apoptosis, are generally observed in WS MSC. Over time, these changes are expected to lead to MSC exhaustion, which will adversely affect further cell expansion and terminal differentiation. In agreement, impaired trilineage differentiation of MSCs to chondrocytes, adipocytes, and osteocytes has been demonstrated in cultured WS MSC [185]. However, it remains unclear how the dysfunction of WRN helicase specifically affects mesodermal differentiation.

Atherosclerosis, which is also common in WS, is primarily caused by endothelial dysfunction. To gain insight into the pathogenesis of atherosclerosis in WS, Ogata and their team revealed that the calcification of subcutaneous tissue in WS patients, resulting in refractory skin ulcers, was attributed to the aging of lymphatic vessels [186]. The calcification of lymphatic vessels and lymphatic endothelial cell dysfunction are consistently observed in WS patients. Laarmann and their team demonstrated that WRN plays a crucial role in maintaining endothelial cell homeostasis. In their study, the knockdown of WRN destabilized the endothelial barrier, compromised HUVEC migration, and increased Ca^2+^ release [187]. These alterations may contribute to the chronic inflammatory process in the endothelium. Furthermore, endothelial cells are influenced by stromal cells, such as pericytes (an MSC-like cell), that help stabilize blood vessels. The loss of WRN in MSC alters the expressions of various cytokine genes [188]. Cytokines that promote angiogenesis are generally downregulated, diminishing the ability of HUVECs to form vessel-like structures [189]. Takayama and Yokote’s team developed a co-culture system that integrates iPSC-derived vascular endothelial cells, macrophages, and vascular smooth muscle cells to replicate atherosclerosis in WS. Using this model, they found that the loss of WRN in macrophages activated type I interferon signaling, leading to increased proliferation of vascular smooth muscle cells. Conversely, endothelial cell proliferation decreased, resulting in atherosclerosis-like characteristics [190]. These recent studies enable us to better understand the disease pathogenesis by dissecting the interactions between different cell types within a defined system. The same mutation in the WRN helicase could directly affect endothelial cells or indirectly affect them through the paracrine actions of other cell types.

As the *WRN* gene is highly similar between human and mouse, Lebel and Leder’s team have generated a progeroid animal model by inactivating the Wrn helicase in mice. The first reported *Wrn* mutant mouse was the *Wrn^Δhel/Δhel^*, which has the RecQ helicase domain deleted. These mutant mice exhibit reduced embryonic survival and ~17% reduction in lifespan among those that survive. Cultured cells from this mutant mouse show some phenotypes similar to those seen in human WS cells, including pro-oxidative status and genomic instability, sensitivity to topoisomerase inhibitors, and reduced replicative potential [191]. Interestingly, the *Wrn*-null mutant mice, despite lacking the full-length Wrn protein, do not display obvious progeroid phenotypes [192]. Thus, the *Wrn^Δhel/Δhel^* and *Wrn*-null mice are crossed with other mutant strains, including *Safb1*-null, *p53*-null, *p21*-null, and *Parp1*-null mice, to investigate the cooperative function of WRN with other DNA repair or cell cycle proteins [192–195]. In the *p53*-null background, *Wrn^Δhel/Δhel^* mice exhibit increased tumorigenicity; however, no such effect is observed in the *p21*-null background [195]. *Parp1* and *Wrn* double mutant mice show a higher frequency of chromatid breaks, complex chromosomal rearrangements, and fragmentation, similar to what is observed in human WS fibroblasts [194]. Additionally, the *Wrn*^−/−^* Terc*^−/−^ double mutant mice exhibit age-related osteoporosis, reduced lifespan, and other progeroid-like characteristics [196]. These studies suggest that the WRN helicase plays a critical role in protecting against genomic instability by interacting with other proteins.

In addition to rodent models, the function of the *WRN* gene has been studied in various other animals, including nematodes, fruit flies, and zebrafish. In *Caenorhabditis elegans*, the WRN homolog acts as a DNA checkpoint; its deficiency leads to reduced lifespan, progeroid tissue phenotypes, increased DNA damage, and genome instability [8, 197–201]. Similarly, the *Drosophila* model of WS exhibits accelerated aging phenotypes and a reduction in lifespan that cannot be reversed by calorie restrictions, which is shown to regulate the intrinsic aging processes via cellular and metabolic adaptations [202, 203]. Other groups including ours have utilized zebrafish as a vertebrate model to investigate the fish *wrn* gene. Knockout of *wrn* in zebrafish displays defects in skeletal development and adipogenesis, which are regulated by pathways similar to those involved in human stem cell differentiation [119, 164, 204, 205]. Using zebrafish as an aging model for drug screening, recent findings from Ma and their team suggest sapanisertib as a potential drug to ameliorate the aging phenotypes effectively [206]. These different animal models combine genetic relevance with physiological complexity, enabling a comprehensive understanding of the pathological mechanisms of WS. Each model, including the human, has its advantages and limitations. Nonetheless, the findings from these studies provide valuable insights into disease development mechanisms and potential therapeutic interventions.

## RECQL4 dysfunction and associated diseases

Unlike BLM and WRN, which are linked to specific diseases with similar symptom clusters, mutations of the *RECQL4* gene can lead to a variety of conditions, including Rothmund-Thomson syndrome (RTS), Baller-Gerold syndrome (BGS), and RAPADILINO syndrome, depending on the specific mutation involved [207]. These three *RECQL4*-associated disorders share some overlapping clinical features, such as developmental defects (e.g., small stature), gastrointestinal disturbances, and radial ray anomalies (Table 2). Further clinical characterization and genetic testing are essential for classifying these conditions as distinct diseases. This scenario illustrates allelic heterogeneity, demonstrating that mutations in a single gene can result in different diseases and various genotype-phenotype correlations, underscoring the critical role of *RECQL4* in human health.

RTS is an autosomal recessive disorder. It is the most well-known condition linked to mutations in the *RECQL4* gene. First described by Rothmund in a Bavarian village during the 1860s, this condition was identified much earlier than the *RECQL4* gene was discovered. Thomson later reported additional cases with similar clinical features and named the syndrome.

RTS is distinct among conditions associated with *RECQL4* mutations as it presents with symptoms of premature aging, sparse hair, and an increased risk of cancer – similar to those seen in WS. The link between RTS and the *RECQL4* mutation was established in 1999 [208]. However, only about two-thirds of RTS patients have mutations in *RECQL4* and are classified as RTS type II, typically presenting with congenital bone defects. The remaining one-third of patients have mutations in *ANAPC1* or in yet unidentified genes that are classified as RTS type I [209]. The most common mutations observed in the type II RTS patients are frameshift or nonsense mutations, which broadly impair or completely eliminate the functionality of the RECQL4 helicase domain [210–213].

BGS is also an autosomal recessive disorder that was first described by Baller in 1950 and later defined by Gerold [214, 215]. The most common cause of BGS is a C-terminal missense mutation in the *RECQL4* gene. Although BGS shares the symptoms such as poikiloderma with RTS, it is specifically associated with coronal craniosynostosis. Additionally, mutations in other genes, such as *FGFR2* and *TWIST*, have also been linked to BGS [216–219].

In contrast to the previous two syndromes, RAPADILINO is not named after the person who characterized it. Instead, the name is a short form of the clinical features of patients presenting RAdial ray malformations, absence of PAtellae, DIarrhea, LImb abnormalities and slender Nose [220]. Unlike RTS and BGS, RAPADILINO syndrome is solely caused by mutations in the *RECQL4* gene. The disruption of the nuclear localization signal (NLS) domain and splicing-site mutations are the most common types of *RECQL4* mutations associated with RAPADILINO. Interestingly, some *RECQL4* mutations linked to RAPADILINO may leave the helicase domain intact [221, 222].

Loss of RECQL4 is also studied in animal models. The original knockout of *Recql4* in mice is embryonically lethal at stages E3.5 to E6.5. As a result, alternative models, such as Recql4-deficiency model, conditional knockout model, and helicase malfunction model, have been developed to replicate the effects of RECQL4 dysfunction observed in patients with specific mutations or loss-of-function alleles [223]. These models allow for a better understanding of the pathophysiology associated with RECQL4 deficiencies and facilitate the exploration of potential therapeutic interventions.

In a study involving such a mouse model, it was found that the deficiency of RECQL4 increases senescence, aligning with the findings in human cells [132]. Furthermore, the role and mechanism of RECQL4 in bone development were demonstrated using a conditional knockout model. In this model, inactivation of p53 led to a rescue of the developmental defects [224]. More precisely, somatic deletion of *Recql4* in murine models has illustrated the critical role of this gene in hematopoiesis. The absence of Recql4 results in accelerated bone marrow failure, which affects various blood cell lineages and increasing apoptosis in multipotent progenitor cells. These findings highlight the importance of RECQL4 in maintaining hematopoietic integrity [225]. Additionally, the roles of RECQL4 in the initiation of replication and maintenance of genome integrity are also demonstrated in *Xenopus* and *Drosophila* models [213, 226–230].

In conclusion, diseases related to RECQL4 dysfunction can be differentiated based on their clinical features. For instance, RTS presents with more developmental defects and an elevated risk of cancer, while BGS exhibits distinct characteristics related to cranial and skeletal development. Additionally, RAPADILINO syndrome is marked by more severe abnormalities in the limbs and gastrointestinal tract.

## CONCLUSION AND FUTURE PROSPECT

In conclusion, all members of the RecQ helicase possess a conserved helicase core formed by two similar domains, which enables them to unwind the structure of nucleic acid duplexes. They play a crucial role in maintaining genome stability through unwinding various secondary structures and recruiting binding partners to aid in replication, transcription, and DNA repair. The severe genetic disorders caused by the mutation of RecQ helicases, including BS, WS, RTS, BGS, and RAPADILINO syndrome, highlight the essential function of RecQ helicases in the maintenance of genome stabilities. Despite multiple animal and cell models being used in the study of RecQ helicases, many questions remain unanswered. Because the RecQ helicases often exhibit overlapping yet distinct functions in genome maintenance, the content-dependent function of each helicase needs further study. Although similar protein domains are thought to perform similar functions, such as the ability of RQC domain to resolve G4 structures, the substrate preferences of each helicase differ significantly. This aspect of uncertainty needs to be further addressed through molecular experiments. Moreover, while significant progress has been made in developing small-molecule inhibitors targeting RecQ helicases for cancer therapy, optimizing these treatments to minimize off-target effects continues to be a challenge. The development of advanced animal models and high-resolution structural analyses will be crucial in uncovering novel therapeutic targets and intervention strategies. Investigating the role of RecQ helicases in age-related diseases and metabolic disorders could yield new insights into their broader physiological functions. Ultimately, integrating multi-omics approaches and innovative gene-editing techniques will pave the way for precision medicine strategies tailored to RecQ-associated diseases.
